# Intra-arterial fibrinolysis for the management of acute ischemia on a below-knee amputation stump. Case report

**DOI:** 10.1590/S1679-45082017RC4014

**Published:** 2017-10-23

**Authors:** Breno Boueri Affonso, Joaquim Maurício da Motta Leal, Rafael Noronha Cavalcante, Priscila Mina Falsarella, Francisco Leonardo Galastri, Rodolfo Souza Cardoso, Felipe Nasser

**Affiliations:** 1 Hospital Israelita Albert Einstein, São Paulo, SP, Brazil.; 2 Faculdade de Medicina de Itajubá, Itajubá, MG, Brazil.

**Keywords:** Fibrinolysis, Ischemia, Amputation stumps, Case reports, Fibrinólise, Isquemia, Cotos de amputação, Relatos de casos

## Abstract

Preservation of the knee joint has enormous advantages in terms of mobility and rehabilitation of an amputee. Any cause of breakdown requiring revision to an above-knee amputation is a major setback because it reduces the patient’s rehabilitative potential. We report a case of intra-arterial thrombolysis use to save a below-knee amputation stump with acute ischemia. A 56-year-old man who sought the emergency department with 1-day history of acute pain on his right below-knee stump. The angiography confirmed popliteal artery occlusion. Pharmacomechanical thrombectomy, with Aspirex (rotational catheter to restore blood flow in occluded vessel, by removing occlusion material from the vessel) and recombinant tissue plasminogen activator, was performed. After 9 years of follow-up the patient remained asymptomatic, capable of independent ambulation with prosthetic limb. Intra-arterial fibrinolysis seems to be a safe and effective treatment for cases of acutely ischemic amputation stump.

## RESUMO

A preservação da articulação do joelho tem grandes vantagens para a mobilidade e a reabilitação de um amputado. Qualquer causa que exija revisão para uma amputação acima do joelho é um grande revés, porque reduz o potencial de reabilitação do paciente. O objetivo aqui foi descrever o uso de trombólise intra-arterial para salvar um coto de amputação abaixo do joelho com isquemia aguda. Homem, 56 anos, procurou pronto atendimento de nosso hospital com histórico de 1 dia de dor aguda em seu coto de amputação infrapatelar direito. A angiografia confirmou oclusão da artéria poplítea. Foi realizada trombectomia farmacomecânica com Aspirex (cateter rotativo para restabelecer o fluxo sanguíneo em vasos ocluídos, removendo material de oclusão do vaso) e ativador do plaminogênio tecidual recombinante. Após 9 anos de seguimento, o paciente permanecia assintomático, capaz de deambulação independente com membro protético. A fibrinólise intra-arterial parece ser um tratamento seguro e eficaz para casos selecionados de coto de amputação com isquêmica aguda.

## INTRODUCTION

The principle of improving the inflow to achieve a lower level of amputation is well known. Preservation of the knee joint has enormous advantages in terms of rehabilitation of an amputee.^(^ ^1^ ^)^ Any cause of breakdown requiring revision to an above-knee amputation is a major setback, as it reduces the patient’s rehabilitative potential because of the lower stability and the highest energetic cost of a gait with above-knee amputees.^(^ ^2^ ^,^ ^3^ ^)^

Acute lower limb ischemia is one of the most challenging conditions in vascular surgical practice and carries a high risk of amputation and death if not treated. Intra-arterial thrombolytic therapy can be considered standard of care for acute peripheral arterial occlusions. It can reclaim arterial perfusion and identify suspicious lesions that can be treated with endovascular techniques.^(^ ^4^ ^)^

This paper describes the use of intra-arterial thrombolytic therapy with recombinant tissue plasminogen activator (rTPA) for the revascularization of a below-knee amputation stump with acute ischemia.

## CASE REPORT

A 56- year-old man sought our service with 1-day history of acute pain on his right below-knee amputation stump. He had undergone right below-knee amputation for critical limb ischemia secondary to trauma 3 years before. After surgery, he had excellent rehabilitation and independent ambulation with prosthetic limb.

Upon clinical examination he had normal femoral pulse, absent popliteal pulse, decreased temperature and cyanosis in the distal third of the below-knee amputation stump. Doppler scan showed above-knee popliteal artery occlusion.

Heparin was administered (80UI/Kg), and right lower limb angiography and catheter directed thrombolysis, with Aspirex^®^ (rotational catheter to restore blood flow; Straub Medical AG, Wangs, Switzerland), were carried out, in the attempt to save the below-knee amputation stump.

**Figure 1:**
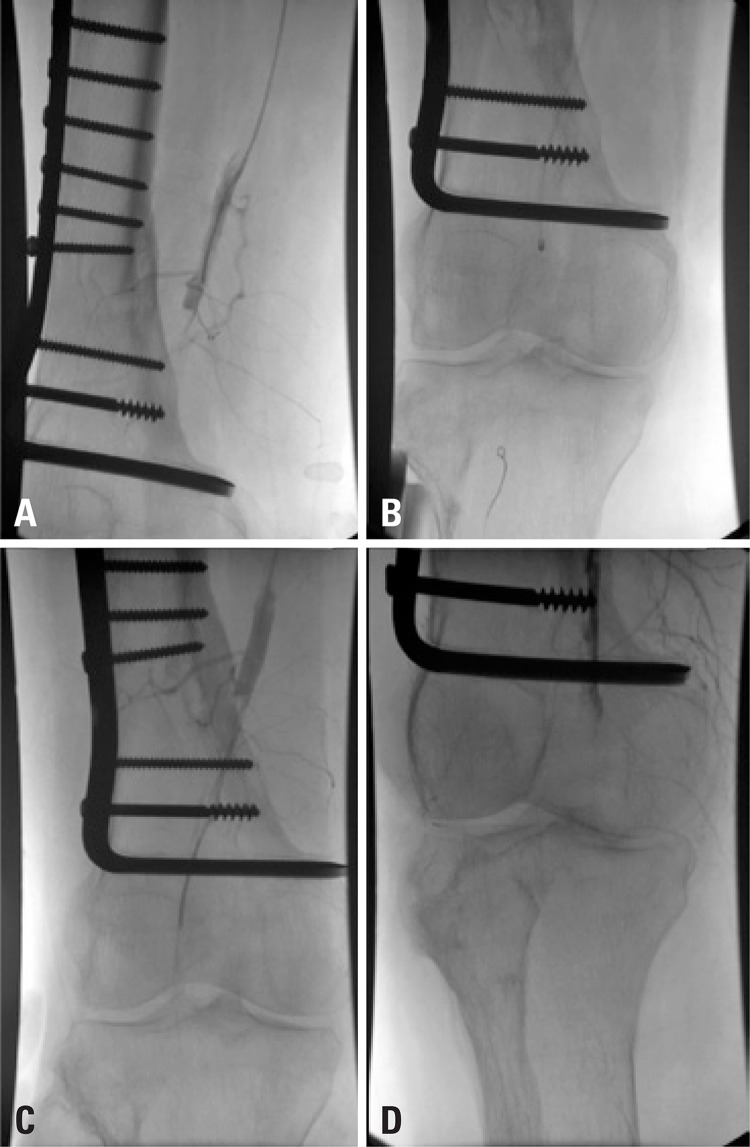
Digital subtraction angiography. (A) Popliteal artery occlusion. (B and C) Thrombectomy catheter placed intra-thrombus. (D) Partial popliteal artery recanalization

Right limb angiography showed superficial and deep femoral arteries without lesions and confirmed popliteal artery occlusion (Figure 1A). A 0,014” guide wire was passed through the occluded area, and a thrombectomy catheter was placed intra-thrombus (Figure 1B). An initial bolus dose of 10mg of rTPA was administered and mechanical thrombectomy was performed with Aspirex^®^ (Figure 1C). Angiography showed partial popliteal artery recanalization (Figure 1D).

**Figure 2:**
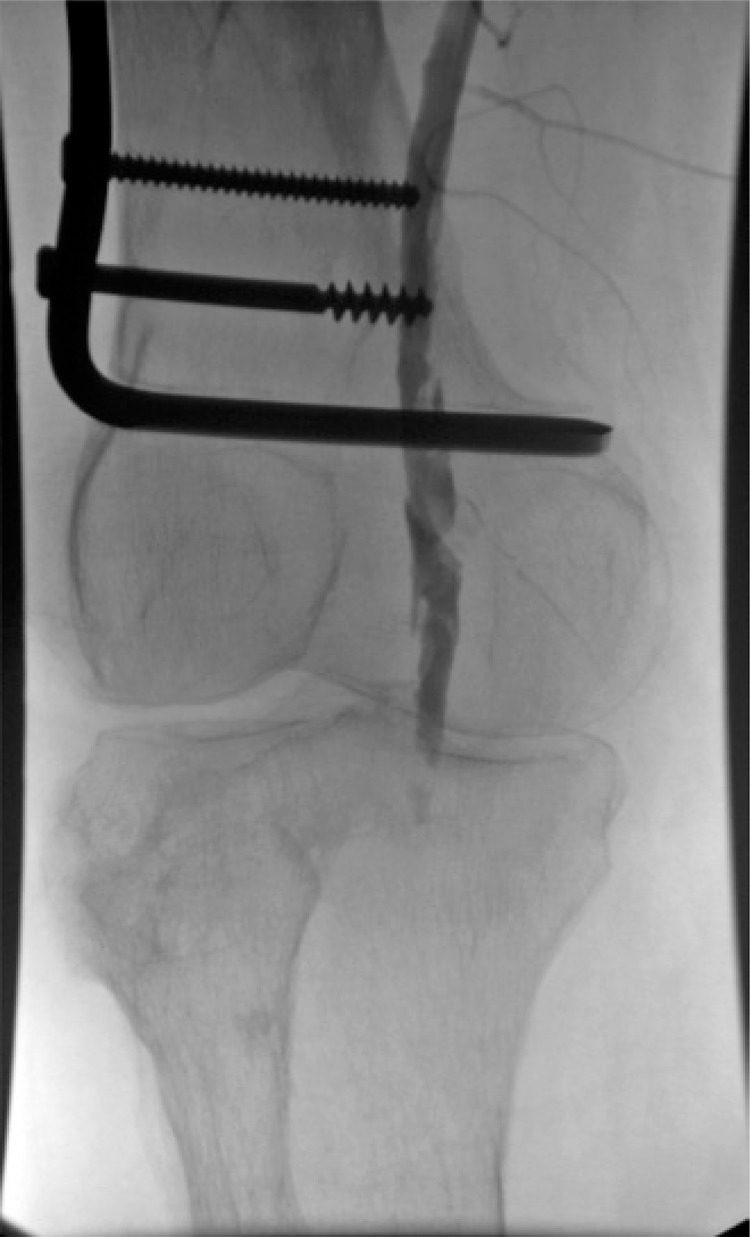
Digital subtraction angiography. Patency of the popliteal artery with residual stenosis and thrombus

**Figure 3:**
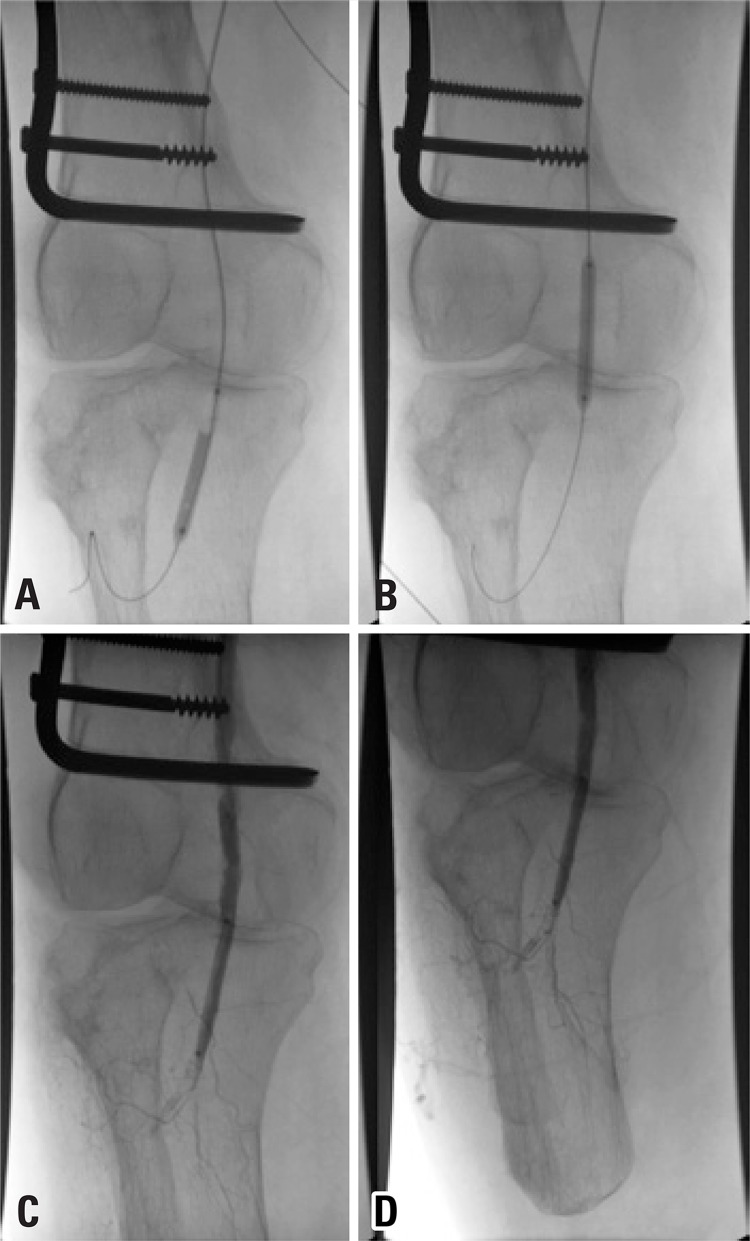
Digital subtraction angiography. (A and B) Popliteal artery angioplasty performed with a 4x20 Paseo-35 balloon. (C and D) Partial popliteal recanalization

**Figure 4:**
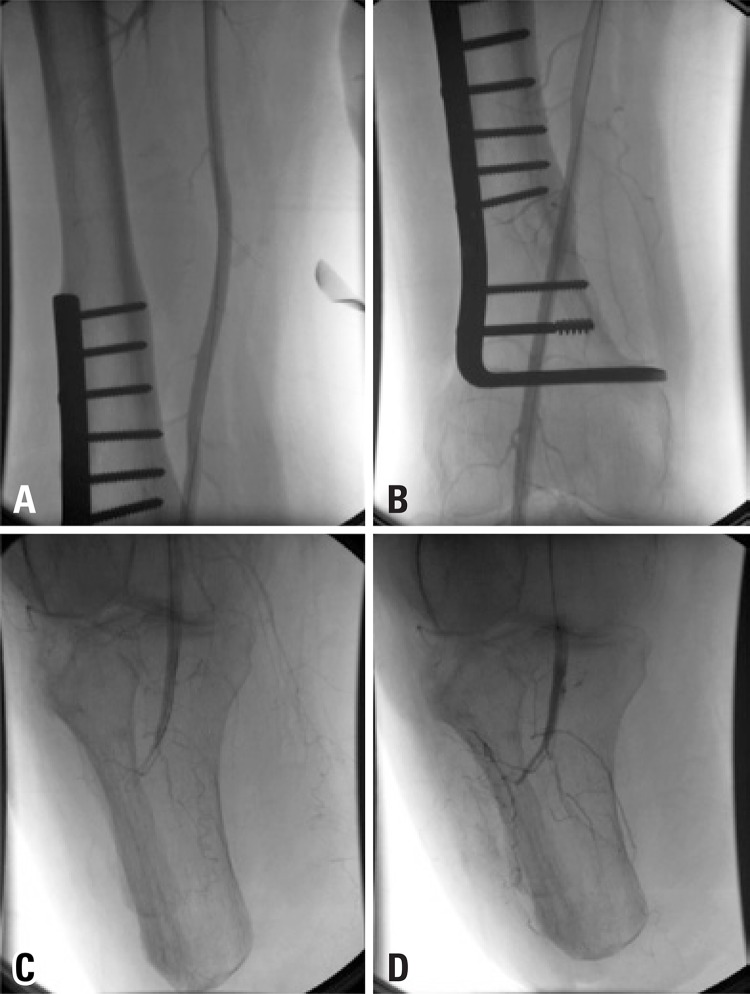
Final angiography demonstrated patency of the superficial femoral and popliteal arteries, without residual stenosis or thrombus

The thrombectomy device was replaced for a multiperforated catheter, and the patient was referred to the intensive care unit to continue rTPA infusion at dose of 3mg/hour and heparinization in full dose (16UI/kg/hour) for 8 hours under close monitoring. A new angiography showed patency of the popliteal artery with residual stenosis and thrombus (Figure 2). Angioplasty was performed with a 4x20 Paseo-35 balloon (Biotronik, Bulach, Switzerland), followed by injection of 10mg rTPA (Figure 3A and 3D). Final angiography demonstrated patency of the popliteal artery, without residual stenosis or thrombus, and excellent inflow to the below-knee amputation stump (Figures 4A and 4D).

The patient had remission of symptoms and was discharged after 5 days. After recovery, he was able for independent ambulation with his prosthetic limb. After 9 years of clinical follow-up the patient remained asymptomatic.

## DISCUSSION

The importance of maintaining the highest number of joints in an amputee patient is mainly due to the complex mechanism needed to keep stability. In elderly patients who already have a lower stability due to loss of muscle mass and osteopenia, to maintain as highest as possible number joints is benefic for the rehabilitation process, which is more difficult among older individuals.^(^ ^3^ ^)^

Despite the technical progress in vascular surgery, acute peripheral arterial occlusion is still characterized by high rates of morbidity and mortality.^(^ ^5^ ^)^ The management of this condition includes anticoagulation, surgical revascularization, and pharmacomechanical thrombectomy, depending on the clinical condition of the patient.

So far, there is no standard of care for management of acutely ischemic stump. Bunt described five cases of ascending gangrene followed by amputation that underwent immediate surgical revascularization. The overall mortality rate of these patients was high (60%).^(^ ^6^ ^)^

In order to avoid stump gangrene, inflow revascularization prior to amputation is usually recommended when there is severe vascular disease, particularly when occlusion of the iliac or common femoral artery exists in combination with occlusion or stenosis of the deep femoral artery. In that sense, Wolosker et al., described the use of intraoperative stenting through the open end of the fibular artery to improve the inflow and achieve a lower level of amputation.^(^ ^7^ ^)^

To our best knowledge, there are only two previous reports on below-knee ischemic stump revascularization for preservation of the amputation level, none of them with thrombolysis.^(^ ^2^ ^)^ Karkos et al., described a femoropopliteal subintimal recanalization of an ulcerated stump in a previously rehabilitated patient. The authors described that the patient was pain-free and able to mobilize at 6-month follow-up.^(^ ^2^ ^)^ Warner et al., described a deep femoral artery angioplasty in a patient with bellow-knee amputation not rehabilitated, presenting pain at rest and non-healing stump wound. After surgery, pain symptoms and wound healing improved significantly.^(^ ^8^ ^)^

In our case, we decided to intervene because patient had a severely ischemic stump, pain at rest and cyanosis. If revascularization were not achieved, the only alternative would be an above-knee amputation. In our service, we usually decide for endovascular approach first and then, if there is failure, we choose open surgical revascularization. In literature, catheter based thrombolysis achieves success in 75 to 92% of cases.^(^ ^9^ ^)^

There is no consensus in literature on the fibrinolytic dose and infusion duration but it is well established that best results are seen in cases with less than 2-week duration.^(^ ^10^ ^)^ In our practice, infusion rates of 0.5 to 5mg/hour are usually chosen for arterial thrombolysis, usually with a control angiography being performed after 6 to 12 hours. It is known that catheter-directed infusion achieves higher limb salvage rates (about 80%) than intravenous systemic therapy (45%) and that the method of fibrinolytic agent delivery (infusion, pulse spray, and dose rate) does not influence limb salvage rates.^(^ ^10^ ^)^

Van den Berg in a review article concluded that there is no difference between surgery and thrombolysis, in terms of mortality and amputation, for acute peripheral arterial occlusion of the lower limb.^(^ ^4^ ^)^ Although the use of catheter-directed thrombolysis is reasonably safe and effective, the risk of bleeding remains. Complications reported include major hemorrhage (5.1%), minor hemorrhage (14.8%), and distal embolization (<1%).^(^ ^4^ ^)^

## CONCLUSION

In selected cases, intra-arterial fibrinolysis seems to be a safe and effective treatment for acutely ischemic amputation stump.
